# Association between the JC Polyomavirus Infection and Male Infertility

**DOI:** 10.1371/journal.pone.0042880

**Published:** 2012-08-13

**Authors:** Manola Comar, Nunzia Zanotta, Eleonora Croci, Immacolata Murru, Roberto Marci, Cecilia Pancaldi, Ornella Dolcet, Stefania Luppi, Monica Martinelli, Elena Giolo, Giuseppe Ricci, Mauro Tognon

**Affiliations:** 1 Institute for Maternal and Child Health - IRCCS “Burlo Garofolo”– Trieste, University of Trieste, Trieste, Italy; 2 Institute for Maternal and Child Health - IRCCS “Burlo Garofolo”– Trieste, Trieste, Italy; 3 Section of Obstetrics and Gynaecology, School of Medicine and Surgery, University of Ferrara, Ferrara, Italy; 4 Section of Cell Biology and Molecular Genetics, School of Medicine and Surgery, University of Ferrara, Ferrara, Italy; University Hospital San Giovanni Battista di Torino, Italy

## Abstract

In recent years the incidence of male infertility has increased. Many risk factors have been taken into consideration, including viral infections. Investigations into viral agents and male infertility have mainly been focused on human papillomaviruses, while no reports have been published on polyomaviruses and male infertility. The aim of this study was to verify whether JC virus and BK virus are associated with male infertility. Matched semen and urine samples from 106 infertile males and 100 fertile males, as controls, were analyzed. Specific PCR analyses were carried out to detect and quantify large T (Tag) coding sequences of JCV and BKV. DNA sequencing, carried out in Tag JCV-positive samples, was addressed to viral protein 1 (VP1) coding sequences. The prevalence of JCV Tag sequences in semen and urine samples from infertile males was 34% (72/212), whereas the BKV prevalence was 0.94% (2/212). Specifically, JCV Tag sequences were detected in 24.5% (26/106) of semen and 43.4% (46/106) of urine samples from infertile men. In semen and urine samples from controls the prevalence was 11% and 28%, respectively. A statistically significant difference (p<0.05) in JCV prevalence was disclosed in semen and urine samples of cases *vs.* controls. A higher JC viral DNA load was detected in samples from infertile males than in controls. In samples from infertile males the JC virus type 2 strain, subtype 2b, was more prevalent than ubiquitous type 1. JCV type 2 strain infection has been found to be associated with male infertility. These data suggest that the JC virus should be taken into consideration as an infectious agent which is responsible for male infertility.

## Introduction

Sexually transmitted infectious agents are considered to be one of the main cause of human infertility. Bacterial agents such as *Chlamydia trachomatis* and *Neisseria gonorrhoeae*, which infect sexually active young adolescents at high prevalence, are considered to be common risk factors for infertility as well as *Treponema pallidum* [1]–[3]. Recent investigations on the putative role of viral agents in infertility and reproduction defects/pathologies have been primarily focused on human papillomaviruses (HPV). [4] Herpes simplex viruses (HSVs), Adenoviruses (AVs), Hepatitis B virus (HBV) and HIV have also been investigated [5]–[8].

The BK (BKV) and JC (JCV) polyomaviruses were discovered in 1971 [9]. BKV was isolated from the urine of a kidney-transplanted patient, while JCV was detected in the brain sample of a patient affected by progressive multifocal leukoencephalopathy (PML), a rare demyelinating disease associated with impaired immunity [9]. BKV and JCV infections are also associated with haemorrhagic cystitis, and chronic meningoencephalitis, respectively. Soon after their isolation, it turned out that BKV and JCV were ubiquitous and infect a large proportion of humans all over the world. BKV primary infection occurs in childhood [9]. At 3 years of age, BKV antibodies are detected in 50% of children, while almost all individuals appear to be infected by the age of 10. JCV primary infection occurs later. Seroconversion is observed at highest rates during adolescence and continues at a lower frequency until the age of about 60, when 50 to 75% of adults show serum antibodies against JCV. Seroepidemiologic data, while essentially confirming previous results, indicate that age-specific seroprevalence is different for BKV and JCV. In fact, BKV seroprevalence reaches 91–98% at 5–9 years of age, whereas JCV seroprevalence varies between 50% and 72% in people over 25 [9]. BKV and JCV primary infections are generally unapparent and rarely associated with clinical diseases. BKV and JCV establish persistent or latent infection which may be reactivated mainly in immunosuppressed, but also in immunocompetent individuals [9]. BKV and JCV are frequently isolated from urine samples. Indeed, the kidney is considered as the main site of BKV and JCV latency in healthy people [9]. BKV and JCV sequences have been detected in peripheral blood mononuclear cells (PBMC), suggesting that infection of blood cells may represent the route by which the virus is spread from the portal of entry to other tissues in the infected host [9]. The modalities of inter-human virus transmission have at present not been completely clarified. The detection of BKV and JCV in tonsils suggest that the two polyomaviruses may be transmitted by the respiratory route [9], [10]. JCV DNA has been revealed in the gastrointestinal tract, whereas BKV and JCV DNA sequences and virions have been detected in raw urban sewage [11], [12]. These data suggest that the oral-fecal route is also a mode of transmission for these viral agents. The presence of BKV and JCV footprints in PBMC, prostate, uterus, kidney, stool, urine and sperm samples also points to haematic, oral-fecal, urine and sexual routes as possible means of polyomavirus transmission in humans [9], [10].

In Europe, the frequency of JCV urine excretion varies between 10% and 46% of individuals. In Asia, the reported frequency is between 13% and 49%, while an American study has reported 41% of JCV excretion. The frequency of BKV excretion in healthy individuals as reported by different studies is negligible [13]. A molecular analysis of JCV types excreted by healthy individuals in Italy, while confirming a high prevalence (46%) of JCV in urine samples, indicated that types 1 and 4 were frequent, whereas type 2 and 3 were very rare [14], [15]. Point mutations in the genes encoding the major viral capsid protein, VP1, is considered sufficient to define the eight JCV types/genotypes and the different related subtypes [16]. The relation between the different JCV types/subtypes and any pathogenicity is still a matter of active investigation. Indeed, a statistically significant association of PML has been shown for JCV type 2, subtype B [17]. In addition, an association between PML and mutations in JCV protein VP1, which change the viral receptor specificity has been reported [18]. However, Sala et al., [19] did not find any correlations between JCV neurovirulence and VP1 polymorphisms. Four different BKV types/genotypes have been isolated. BKV genotype I (BKV-I) is considered the main type circulating in humans worldwide, while BKV genotypes II, III, and IV are less prevalent. Early studies detected polyomavirus (PYV) footprints in sperm fluids from healthy individuals [20]–[22]. In these investigations, BKV sequences were detected by PCR in 18/19 [20], 18/20 (90%) [21], and in 5/6 (83%), while the same 6 samples were also JCV negative [22]. One limitation of these studies is the small sample size [20]–[22]. In prostate cancers, BKV DNA sequences were detected at high prevalence ranging from 44% of the viral regulatory region, detected by PCR [23], to 58% of samples found BKV-DNA positive by Southern blot hybridization [24].

To date, no studies have been reported on the association between human polyomaviruses and male infertility.

The aim of this study was to investigate if the presence of JCV and BKV DNA in semen and urine samples is associated with male infertility.

## Materials and Methods

### Samples

Paired semen (n = 106) and urine (n = 106) samples were collected from infertile male subjects (median age, m.a., of 37.0±6 years), during 2011, from discarded specimens (n = 212) from our Clinical Laboratories after spermiogram and other routine analyses. Anonymously collected samples from males were coded, with indications of age, and pathologies if any. The semen samples, with altered sperm parameters, were from males who had declared more than one year without conception, without any clinically apparent HPV lesions/infections. The males, all of whom were of Caucasian origin living in the same urban area, had not been hospitalized within the past two years. They were not assuming chronic systemic medication; were without seminal bacterial infections or testicular trauma, and were considered “well” on the day of the sample collection. Controls were 100 semen and urine samples from fertile males (m.a. 36.2±4 years). Semen was collected by masturbation after 3 days of sexual abstinence. The semen spermiogram analysis, for infertility evaluation, including volume (<2 ml), concentration (<20 million sperm/ml), motility (<50%) and morphology (<30%) of spermatozoa, was performed according to the World Health Organization (WHO) guidelines 1999 [25], which are more restrictive than the new WHO guidelines issued in the 2010 [26]. The study was approved by the Institutional Scientific Board of the Burlo-Garofolo Children’s Hospital, Trieste. Informed written consent was obtained from the subjects.

### DNA Extraction

DNA was isolated from 400 µl of semen and 400 µl of urine samples and eluted in a total volume of 100 µl using a commercial kit (High Pure PCR Template preparation Kit, Roche Applied Science, Mannheim, Germany), as indicated by the supplier. DNA was stored at −80C° until the time of analysis.

### Viral DNA Load

Specific quantitative real-time PCR assays, using TaqMan chemistry, were performed for JCV and BKV DNA detection using the AB PRISM 7000 Sequence detection System (Applied Biosystems, Milan, Italy). A multiple quantitative real-time PCR (Q-RT) was designed to simultaneously amplify the large T antigen (Tag) coding sequences of BKV and JCV, and the cellular ß-globin reference gene sequences [10], [27], [28]. For each reaction run, 10 µl of DNA from clinical samples, and 10 µl of the specific standard scale dilution, from 10^7^ to 10^0^ copies, were added to a final volume of 50 µl of reaction-mix. The corresponding copies/ml of semen/urine samples were 1×10^9^ copies/ml and 100 copies/ml, respectively.

Tight precautions were taken to avoid cross-contamination. Separate rooms were used to extract the nucleic acids, to prepare the amplification mixtures, and to run the Q-PCR. Multiple negative controls containing distilled sterile water, void of DNA templates, were included in each Q-PCR batch.

### Viral DNA Sequence Analysis

**Figure 1:**
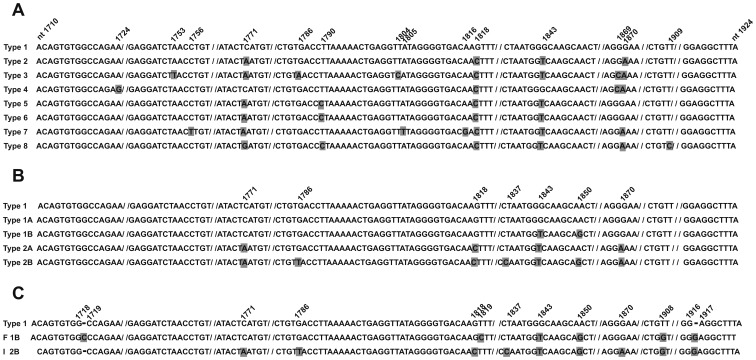
Alignment of JCV VP1 sequences, nt 1710–1924, from fertile (F) and infertile (I) subjects. Panel A . The most representative JCV genotype strains, types 1–8 are indicated. Nucleotide substitutions in JCV strains are numbered and marked in grey. **Panel B**. JCV 1 and 2 strains and related subtypes 1A and 1B, and 2A and 2B are indicated. Nucleotide substitutions are numbered and marked in grey. **Panel C**. VP1 coding sequences of JCV 1B and 2B strains indentified in semen and urine samples from fertile (F) and infertile (I) subjects, respectively, compared to JCV 1 strain (upper line). Nucleotide substitutions and insertions are numbered and marked in gray. The sequence homology of JCV genotypes was compared with the Blastn using flat master-slave with identities of the National Centre for Biotechnology Information (NCBI). Sequences used for the analysis include on Panel A: Mad-1 strain of JCV type 1[GenBank, accession no.J02227]; MY strain of JCV type 2[GenBank, accession no.AB038250]; #308 of JCV type 3 [GenBank, accession no.U73500]; #402 of JCV type 4 [GenBank, accession no.AF015528]; #501 of JCV type 5 [GenBank, accession no.AF015684*]; #601 of JCV type 6 [GenBank, accession no. AF015537]; #701 of JCV type 7 [GenBank, accession no. AF295737]; #801 of JCV type 8 [GenBank, accession no. AF281623]. Panel B: Mad-1 strain of JCV type 1; #124 of JCV type 1A [GenBank, accession no. AF015526]; #123 of JCV type 1B [GenBank, accession no. AF015527]; #226 of JCV type 2A [GenBank, accession no. AF015531]; #223 of JCV type 2B [GenBank, accession no. AF015532].^ *^Record removed. *This record was removed at the submitter’s request because the sequence cannot be confirmed.*

In order to genotype the JCV strains, a 215-bp fragment of the VP1 gene was analysed [29]. Specifically, VP1 coding sequences, nt 1,710–1,924, were subjected to PCR amplification using the primer set JLP-15 (5′-ACAGTGTGGCCAGAATTCCACTAC-3′, nt 1710–1734), -JLP-16 (5′-TAAAGCCTCCCCCCCAACAGAAA- 3′, nt 1924–1902, Figure 1). This DNA sequence encodes for significant amino acid residues of the viral capsid protein, VP1 [29]. The polymorphism of these polypeptide domains and their encoding DNA sequences enable eight different JCV types and subtypes (Figure 1A) to be distinguished. PCR amplifications were carried out with technical conditions reported elsewhere [29]. Direct automatic DNA sequencing was performed using the Big dye terminator chemistry v.3, under standard conditions, with an ABI PRISM 310 Genetic Analyzer (Applied Biosystem, Foster City, CA). A sequence homology search was performed using the Basic Local Alignment Search Tool (BLAST) program via the National Centre for Biotechnology Information (NCBI) website [17], [30]. Although males with clinically apparent HPV infection were not enrolled in this study, HPVs were investigated as a control. The HPV sequence was amplified by PCR and genotyped for up to 37 of the most clinically relevant HPVs, that are 13 high-risk and 24 low–risk types, using the Linear Array HPV Detection and Genotyping test (Roche Molecular Systems, Milan, Italy) as described by the manufacturer. The lower limit of assay detection was 76 viral copies/µl of sample [27].

### Statistical Analysis

Categorical data are presented as numbers and percentages. Descriptive statistics were used to describe clinical and laboratory parameters. The association between several possible explanatory variables and study outcomes was explored by logistic regression analysis. A *p* value = <0.05 indicated statistical significance. Data were analysed using SPSS 2001 for Windows 11.0. The T-student test was used for viral DNA load statistical calculation.

## Results

Semen and urine samples from infertile and fertile males, for a total of 412 samples, were investigated by quantitative Real Time PCR for the presence of viral DNA sequences belonging to BKV and JCV genomes.

In the infertile group (n = 212), JCV was identified in 34% (72/212), whereas BKV was present only in 0.94% (2/212) of the samples. Interestingly, in this group, JCV sequences were detected in 43.4% (46/106) of urine and in 24.5% (26/106) of semen samples. The overall prevalence of JCV/BKV infection, in all the examined samples from the infertile group was 34.9% (74/212).

JCV was detected in 19.5% (39/200) and BKV in 0.5% (1/200) of samples from fertile males (n = 200), employed as control group. Specifically, JCV DNA was found in 28% (28/100) of the urine samples, and in 11% (11/100) of the semen samples. Only 1 sample was detected as BKV-positive in 100 urine samples, while none of the 100 sperm samples was found to be BKV-positive (Table 1). In the univariate statistical analysis, JCV prevalence detected in semen samples of cases *versus* their correspondent controls differed significantly (semen *p* = 0.034), while for urine it tended to be significative (urine *p* = 0.076). Since the prevalence of BKV sequences in these samples was negligible, no statistical analysis was performed.

**Table 1:** Prevalence, distribution and viral DNA load of JCV and BKV sequences in semen and urine samples from infertile (I) and fertile (F) males.

Polyomavirus			I				F	
	urine	viral load(m.v.)*	semen	viral load(m.v.)*	urine	viral load(m.v.)*	semen	viral load(m.v.)*
**JC**	46/106(43.4%)	2.0×10^6^copies/ml	26/106(24.5%)	1.1×10^3^copies/ml	28/100 (28%)	7.8×10^5^copies/ml	11/100 (11%)	4.1×10^2^copies/ml
**BK**	2/106(1.9%)	3.5×10^2^copies/ml	0/106(−)	–	1/100(1%)	4.1×10^2^copies/ml	0/100(−)	–

* mean value.

The median age of the infertile males that excreted JCV in their urine was 33.8 ys, while in fertile males it was 38.9 ys. In addition, the median age of infertile males with JCV both in urine and semen samples was 41 ys, whereas no fertile subjects had JCV in both samples.

In samples from infertile males, JCV DNA load quantification showed a constantly high copy number among urine samples, with a mean viral load of 2×10^6^ copies/ml (range: 1×10^2^ copies/ml to 6×10^7^ copies/ml), while the mean JCV DNA load in semen samples was 1.1×10^3^ copies/ml (range: 2×10^2^ copies/ml to 1.8×10^3^ copies/ml).

In contrast, in samples from fertile males the amount of JCV mean viral DNA load in urine samples was 7.8×10^5^ copies/ml (range: 5×10^2^ copies/ml to 7.2×10^6^copies/ml), whereas in semen samples it was lower [mean viral load: 4.1×10^2^ copies/ml (range: 1×10^2^ copies/ml to 5.5×10^2^ copies/ml)].

It should be noted that the amount of JCV DNA was significantly higher in semen (p = 0.001) and in urine samples (p = 0.007) from infertile males compared to control subjects.

A low viral BKV DNA load was measured in the urine from the two BKV-positive infertile males, with a mean viral load of 3.5×10^2^ copies/ml (range from 1.2×10^2^ copies/ml to 5.8×10^2^ copies/ml).

All samples were successfully amplified with ß-globin primers within a range of 45 copies/reaction to 1.6×10^6^copies/reaction.

**Figure 2:**
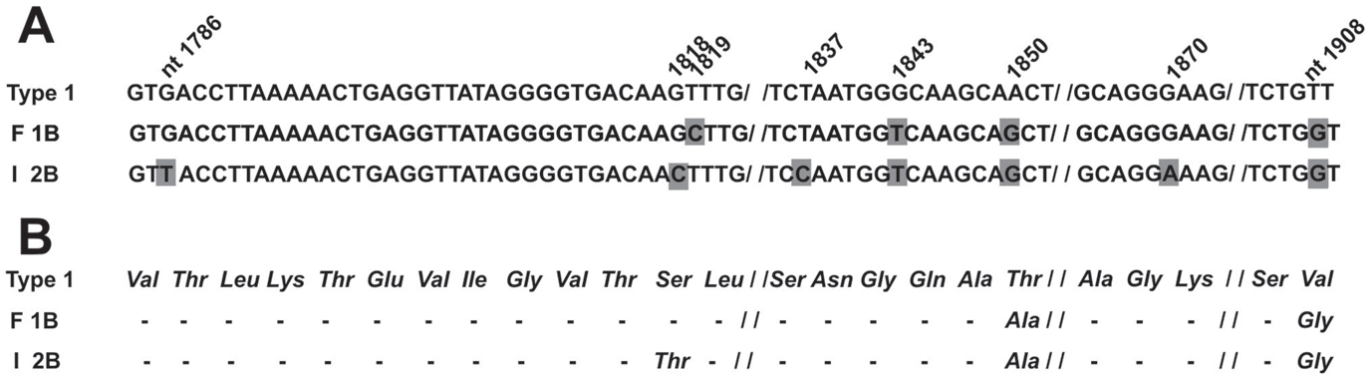
Amino acid substitutions in the VP1 region based on JCV sequences identified in semen and urine samples from fertile (F) and infertile (I) subjects. Panel A. Alignment of JCV VP1 coding sequences, nt 1784–1908, of JCV 1B and 2B subtypes detected in semen and urine samples from fertile (F) and infertile (I) subjects, respectively, compared to JCV type 1 (upper line). **Panel B.** Amino acid substitutions in the VP1 region of JCV subtypes from semen and urine samples from fertile (F) and infertile (I) subjects, respectively, compared to amino acid sequence of JCV type 1 (upper line).

In order to determine the JCV genotype in positive samples from infertile and fertile subjects, DNA sequence analysis was carried out. To this purpose, a specific 215-bp fragment, nt 1,710–1,924 from the VP1 coding sequences, which belongs to the JCV late region (Figure 1A), was PCR amplified and then subjected to automatic DNA sequencing. These DNAs were from urine and semen samples which had been found to be JVC-positive for Tag sequences in the previous PCR analysis. Interestingly, it turned out that the most prevalent JCV strain in urine and semen samples from infertile males (I) was genotype 2b, (AF015532.1) while in samples from fertile individuals (F) the most representative JCV strain was the genotype 1b (AF015527.1) (Figures 1B and 2A). Indeed, JCV genotype or strain 2 was detected in 73% (19/26) of semen samples from infertile males. Interestingly, 73.7% (14/19) of these JCV type 2-positive samples, was genotyped as JCV subtype 2b. Three JCV 2b-positive samples showed (n = 2) a T→G substitution at nt 1,908, and (n = 1) a G insertion at nt 1,916 (Figure 1C, lines F 1B and I 2B; Figure 2A, lines F 1B and I 2B), while amino acid substitutions are shown in Figure 2 B, lines F 1B and I 2B.

In the control group (F), 72.7% (8/11) of the semen samples showed JCV type 1 sequences (Figure 1B). Among these 8 JCV-positive DNA, 5 samples, 62.5% (5/8) carried JCV sequences belonging to subtype 1b (Figure 1B, C; Figure 2A). DNA sequencing showed point mutations which are cumulatively indicated in Figures 1C and 2A.

JCV genotyping of matched urine samples from both groups of subjects confirmed the DNA sequencing data obtained from the semen samples.

It is noteworthy that 84.6% (22/26) of JCV-positive semen samples from infertile males had a significant reduction in motility, while 76.9% (20/26) showed altered morphology. No association was detected between JCV and semen volume and concentration. With regard to semen samples (n = 100) from the control group, only 11% (11/100) were JCV-positive.

Since infertile males, with clinically apparent HPV-infection, were not enrolled in this study, HPV was found, as expected, with a low prevalence in their semen (2%), a rate which did not differ substantially from that detected in semen samples from the control group (1.1%). A similar low HPV prevalence was also revealed in urine samples from both groups.

## Discussion

The role of infections in the pathogenesis of infertility has been recognised. Indeed, infectious agents are responsible for numerous diseases which may cause difficulty in conceiving. Several studies have associated viral infections to infertility. HPV is one of the most highly studied viral agents in male and female infertility. In addition, it has been shown that sperm may function as vectors for HPV transfer in the oocytes, thus altering the progress of pregnancy. [31], [32].

Detection of human polyomaviruses, such as JCV and BKV, has been reported in urinary tract and prostate tissues [20], [23], [33], [34] and in semen samples from males that have undergone spermiogram analysis [20]–[22].

Monini et al. reported detecting BKV sequences by PCR and Southern blotting in more than 50% of both normal and tumour tissues obtained from the urinary tract and prostate [20]. Zambrano et al. found [33] BKV DNA in 3/12 frozen tissue prostate specimens which were subject to analysis (2 cancerous and 1 normal tissues), whereas JCV was detected in 6/12 (50%) of cancerous and in 5/12 normal prostate tissues. Das et al. detected BKV in more than 79% of neoplastic prostate tissues examined by both PCR and ISH [34], whereas in normal prostates the prevalence of BKV DNA was 27% [23]. These molecular epidemiology data indicate that human polyomaviruses are sexually transmitted viral agents. Recent studies have reported on the detection of anti-BKV IgM antibodies in the cord blood of newborns from mothers infected with BKV, while specific viral DNA sequences were revealed in the placenta, kidney and brains of aborted foetuses suggesting possible maternal-fetal transmission of the BKV infection [35].

To date, no data has been published on the prevalence of JCV and BKV infections in semen from infertile males and their association with infertility.

In the present study, BKV DNA was detected at a low frequency in samples from infertile males and from the control group. A high prevalence of JCV sequences were revealed in semen (24.5%) and urine (43.4%) samples from infertile males. In the control, represented by samples from fertile subjects, the prevalence of JCV sequences was low, 11% in semen and 28% in urine samples, respectively. The difference is statistically significant.

The molecular quantification of the viral DNA load in infected urine and semen samples is suggestive of different BKV and JCV replication and reactivation. These characteristics may have implications for BKV and JCV transmission and pathogenesis. In fact, it has been reported that intermittent JCV shedding in the urine of asymptomatic subjects was age-dependent [36].

In our study the two cohorts, infertile and fertile males, had a similar median age. We observed that the median age of the infertile males that excreted JCV in their urine was 33.8 ys, while in fertile males it was 38.9 ys. In addition, the median age of infertile males with JCV both in urine and semen samples was 41 ys, whereas no fertile subjects had JCV in both samples.

JCV sequences were detected both in urine and semen samples with a remarkable higher viral DNA load and prevalence in the cohort of infertile males compared to the age-matched control group, suggesting high susceptibility to virus reactivation/infection and increased multiplication activity. Moreover, JCV and BKV shedding in infertile males appeared to occur independently, as their DNAs were not simultaneously detected in any of the cases studied.

In order to find out whether an association occurred between a specific JCV strain and samples from infertile males, a DNA fragment from the JCV VP1 late region was molecularly characterised in samples found to be JCV Tag-positive. DNA sequence analysis indicates that JCV genotype 2 is significantly more prevalent in samples from infertile males than in those from fertile individuals. Indeed, JCV genotype 1 was the prevalent stain in the control group. This result is in agreement with epidemiologic data reporting JCV genotype 1 as the most common JCV strain circulating in Italy. [14] The significance of the higher prevalence of JCV strain 2b in samples from infertile males deserves more evaluation. The potential higher pathogenicity and virulence of genotype 2, regarding both subtypes a and b, was recently described by different teams in distinct groups of immunocompromised patients [37], [38]. These results strongly support the hypothesis that some JCV genotypes could be involved to a greater extent than others in the pathogenesis process, including that relating to male infertility. Of particular interest are our findings from 14 infertile males who simultaneously showed JCV infection with genotype 2b in urine and semen samples, although a different viral DNA load was observed in these matched samples. It has been suggested that JCV VP1 polymorphisms, which are similar to those detected in our study, may account for some biological characteristics, such as cell tropism, cell binding, virus reactivation and multiplication [18], [39]. In this investigation, no functional assays have been carried out with JCV VP1 variants, consequently no firm conclusion can be drawn on the putative higher pathogenicity of JCV strains detected in semen and urine samples from infertile males.

Taken as a whole, our data suggest an association between JCV infection and male infertility and indicate that JCV may represent a new risk factor which should be considered regarding male infertility. In addition, our data seem to indicate an association between the presence of JCV in semen samples with altered sperm motility and morphology, as previously reported for HPV [40], as well as for other viral agents [41], thus confirming that sperm alterations are often affected by viral infection.

In conclusion, this study indicates for the first time an association between JCV and male infertility. It is worth noting that such an association does not provide proof of the cause of infertility. The particular conditions of the host, such as genetic predisposition, transient impairment of the immune system, specific JCV infection/reactivation may account for the reported data. We may speculate that the presence of JCV in the DNA of semen samples from infertile males may also represent a novelty in the assisted reproduction field and may have implications for the success of egg fertilization and embryo development. Further investigations are necessary to better understand the possible role of JCV in male infertility, including virus localisation in spermatozoa, in exfoliated cells, and the clinical follow-up of subjects with infected semen.
